# Cervical Arthroplasty for Traumatic Disc Herniation: An Age- and Sex-matched Comparison with Anterior Cervical Discectomy and Fusion

**DOI:** 10.1186/s12891-015-0692-1

**Published:** 2015-08-28

**Authors:** Hsuan-Kan Chang, Wen-Cheng Huang, Jau-Ching Wu, Tsung-Hsi Tu, Li-Yu Fay, Peng-Yuan Chang, Ching-Lan Wu, Huang-Chou Chang, Yu-Chun Chen, Henrich Cheng

**Affiliations:** Department of Neurosurgery, Neurological Institute, Taipei Veterans General Hospital, Room 508, 17F, No. 201, Shih-Pai Road, Sec. 2, Beitou, Taipei, 11217 Taiwan; School of Medicine, National Yang-Ming University, Taipei, Taiwan; Institute of Pharmacology, National Yang-Ming University, Taipei, Taiwan; Department of Radiology, Taipei Veterans General Hospital, Taipei, Taiwan; Department of Surgery, Kaohsiung Veterans General Hospital, Kaohsiung, Taiwan; Department of Medical Research and Education, National Yang-Ming University Hospital, I-Lan, Taiwan; Institute of Hospital and Health Care Administration, National Yang-Ming University School of Medicine, Taipei, Taiwan

## Abstract

**Background:**

The efficacy and safety of using cervical arthroplasty for degenerative disc disease have been demonstrated by prospective, randomized and controlled clinical trials. However, there are scant data on using cervical arthroplasty for traumatic disc herniation. Therefore, this study aimed to investigate the outcomes of patients who underwent cervical arthroplasty for traumatic disc herniation.

**Methods:**

This cohort included patients who were admitted through the emergency department for trauma. Only patients who had newly-onset, one- or two-level cervical disc disease causing radiculopathy or myelopathy were identified. None of these patients had previously sought for medical attention for such problems. Those patients who had severe spinal cord injury (i.e. American Spinal Injury Association scale A, B or C) or severe myelopathy (i.e. Nurick scale 4 or 5), bony fracture, dislocation, perched facet, kyphotic deformity, or instability were also excluded. An age- and sex-matched one-to-one comparison was made between patients who underwent cervical arthroplasty, on the one hand, and anterior cervical discectomy and fusion (ACDF).

**Results:**

A total of 30 trauma patients (15 in the arthroplasty group and 15 in the ACDF group) were analyzed, with a mean follow-up of 29.6 months. The demographic data were similar. Post-operation, the arthroplasty group had significant improvement in VAS of neck and arm pain, JOA, and NDI when compared to their pre-operation status. Similarly, the ACDF group also improved significantly after the operation. There were no differences between the two groups in post-operative VAS neck and arm pain, and JOA scores. The arthroplasty group maintained a range of motion in the indexed levels and had better NDI scores at 6-months post-operation than the ACDF group.

**Conclusions:**

For selected patients (i.e. no spinal cord injury, no fracture, and no instability) with traumatic cervical disc herniation, cervical arthroplasty yields similar improvement in clinical outcomes to ACDF and preserves segmental mobility.

## Background

The efficacy and safety of using cervical arthroplasty for degenerative disc disease (DDD) have been demonstrated by several prospective, randomized and controlled studies by the United States Food and Drug Administration Investigational Device Exemption (FDA-IDE) trials [1–10]. These trials enrolled patients with cervical DDD or spondylosis causing radiculopathy, myelopathy, or both, and demonstrated that the clinical outcomes were similar at two to eight years of follow up for both cervical arthroplasty and anterior cervical discectomy and fusion (ACDF) [11]. However, there are scant data in the literature on using cervical arthroplasty for traumatic disc herniation. In the opinion of several experts, cervical trauma causing ligamentous or bony injury has been listed as a contraindication for cervical arthroplasty [12–14]. Nevertheless, it is not uncommon to see patients with symptomatic disc disease after minor neck injury. Although there are inadequate data, cervical arthroplasty may be an option for these patients who had no spinal cord injury, fracture, or instability, but mainly disc disease.

Anterior cervical discectomy has been commonly accepted as the management of neural tissue compression caused by disc herniation, albeit degenerative or traumatic [15–19]. For patients with trauma, ACDF could not only replace the broken disc but also provide immediate fixation by application of plate and screws, whereas cervical arthroplasty provides less immediate stability post-operatively. Therefore, it is clear that traumatic disc herniation coexisting with instability such as fracture, dislocation, or compromised posterior ligamentous complex would definitely require ACDF rather than arthroplasty. On the other hand, in a carefully selected patient—for example, a young person who experienced minor neck trauma while playing sport and who had no instability or facet joint disease but a traumatic disc herniation causing myelo-radiculopathy that warranted anterior cervical discectomy—cervical arthroplasty might be a reasonable alternative to conventional ACDF. There are reports addressing adjacent segment disease (ASD) after ACDF as well as subsequent secondary surgery [20, 21]. Moreover, these male and younger patients who have a higher risk of trauma are also of higher chances of ASD and the re-operations [21]. Therefore, cervical disc arthroplasty might be particularly valuable for these trauma patients if it reduces the development of ASD. Although this potential advantage has not yet been proven by the currently available clinical trials for degenerative disc disease, it is reasonable to expect some differences in the setting of trauma, which could accelerate the pathology of ASD.

This study aimed to investigate the outcomes of patients who underwent cervical arthroplasty for traumatic disc herniation. The FDA-IDE trials comparing arthroplasty to ACDF did not specifically look into these patients.

## Methods

### Study design

Medical records, images and neurological evaluations of our institute were retrospectively reviewed. All patients who had neurosurgical consultation and were admitted through the emergency department of our institute for cervical spinal trauma, from May 2007 to December 2011, were identified. The inclusion criteria were patients with one- or two-level traumatic cervical disc herniation who required and received anterior cervical discectomy and either arthroplasty or ACDF. None of these patients had previously sought for medical attention for such problems. In other words, only those patients who had not had radiculopathy and myelopathy seeking medical attention previously were included. Thus the cervical disc disease was most likely related to the trauma event rather than degeneration. The exclusion criteria were: (1) severe spinal cord injury equal to or worse than the American Spinal Cord Injury Association (ASIA) impairment scale C (i.e. ASIA scale A, B and C); (2) severe cervical myelopathy (i.e. Nurick scale 4 or 5); (3) bony fracture or evident segmental instability (i.e. more than 3.5 mm translation or 20° angular motion) at the indexed level; (4) segmental arthrodesis without mobility; (5) incompetent facet joints;(6) adjacent segment disease after previous cervical fusion; (7) ossification of posterior longitudinal ligament (OPLL), or (8) kyphotic deformity. Patients who underwent previous cervical spine surgery or had disc disease at more than two levels were also excluded.

Each of the patients fulfilling the above mentioned criteria and who underwent one- or two-level cervical arthroplasty was compared to one age- and sex-matched patient who underwent ACDF for a similar condition during the same period of time. All the demographic data, operation notes, peri-operative medical records, clinical outcomes, and radiographic evaluations were analyzed. Written informed consent was obtained from participants, and the Institutional Review Board, Taipei Veterans General Hospital, approved the study.

### Surgical technique

For cervical arthroplasty, the patient was placed in a supine position under general anesthesia. A right-sided horizontal incision along the skin crease in the neck was made correlating to the target level of the cervical disc. Intra-operative fluoroscopy was used to confirm the target level(s). Generous decompression of the bilateral neuroforamen was performed after discectomy with resection of bilateral uncovertebral joints. Resection of the posterior longitudinal ligament was routinely performed on every patient to ensure adequate decompression of the spinal cord. Copious saline irrigation was applied during the whole procedure of drilling of the osteophytes and milling of the endplates. We aimed to achieve optimal carpentry of the cervical arthroplasty by meticulous endplate preparation and appropriate sizing of the artificial disc [22]. One of two kinds of arthroplasty devices, a Bryan disc (Medtronic, Memphis, TN) or Prestige LP (Medtronic, Memphis, TN) artificial disc, was implanted in this series of patients under guidance of intraoperative fluoroscopy. A closed-system drainage catheter was then placed and the wound was closed layer by layer in every patient.

For ACDF patients, the surgical approach and techniques of decompression were very similar to that used for arthroplasty patients. All ACDF procedures used interbody cages and were instrumented with titanium cervical plates and screws.

### Evaluation of clinical outcomes and radiographic studies

Standardized clinical outcomes, including visual analogue scale (VAS), neck disability index (NDI), and Japanese Orthopedic Association (JOA) scores, were collected at each time-point for follow-up post-operation, at approximately 6, 12 and 24 months. Data were collected by two special nurse assistants under the physicians’ supervision during clinic visits.

Standard anterior-posterior/lateral and lateral flexion/extension radiographs were taken at each time-point mentioned above. Radiographic reports and range of motion (ROM) at target level(s) were interpreted and measured using the PACS system software, SmartIris (Taiwan Electronic Data Processing Co., Taiwan) on a medical-use screen by independent radiologists or surgeons. ROM was measured on dynamic lateral radiographs. Angulation of arthroplasty was determined using Cobb criteria as same as one of the FDA-IDE trials [6]. Repeated measurement was undertaken, and a final decision was made by the senior author of this study if there was any discrepancy in the interpretation or measurement.

### Statistical analysis

Independent *t*-tests and paired *t*-tests were used for data analysis using the SPSS Software (SPSS Inc., Chicago, USA). The statistical significant value was defined as *p*-value of <0.05.

## Results

### Identification of patients with traumatic disc herniation

During the study period, only patients who were admitted via the emergency department of our institute for cervical spine trauma were qualified as candidates for further analysis. The surgeons were neutral to both the surgical approaches and provided the patients as well as the family with equally adequate information for both the ACDF and cervical arthroplasty. The eventual choice of ACDF or cervical arthroplasty was made upon their preference. Both types of surgery were equally priced by the National Health Insurance of Taiwan, which has universal coverage. Theoretically, there was little selection bias from the surgeons’ aspect.

A total of 16 patients fulfilled the inclusion criteria described above and underwent one- or two-level cervical arthroplasty. Among them, 15 (94 %) patients completed the scheduled follow-ups and were thus analyzed as the arthroplasty group. For each patient in the arthroplasty group, one age- and sex-matched patient who received ACDF was selected from the cohort for comparison.

### Demographic data

Owing to the specifically tailored comparison (i.e. matching age and gender), there were little differences in the demographic data between both groups (Table 1). Among the 15 patients of the arthroplasty group, there were 11 (73.3 %) males and 4 (26.6 %) females, and the mean age was 48.8 ± 9.3 years. The mean follow-up time was 29.6 ± 9.1 months. The segmental mobility was similar in both groups prior to the operation (mean range of motion:5.3 ± 1.6 and 7.4 ± 3.6, *p* = 0.113, the arthroplasty and the ACDF groups, respectively). Furthermore, both groups had similar rates of underlying medical conditions, including cigarette smoking, diabetes, hypertension, and end-stage renal disease requiring hemodialysis.

**Table 1:** Comparison of the demographic data

	Arthroplasty	ACDF	*p* value
No. of patients	15	15	
Age (years)	48.8 ± 9.3	53.6 ± 12.3	0.234
Gender			
Male	11	11	
Female	4	4	
Mean operation time (min)	214.3 ± 56.8	200.6 ± 58.1	0.52
Pre-op ROM (degree)			
Mean	5.3 ± 1.6	7.4 ± 3.6	0.113
Cigarette smoking	3	3	1.00
Diabetes	2	2	1.00
Hypertension	4	5	0.694
End-stage renal disease	0	0	1.00

*ROM*: range of motion at the indexed levels

The level distribution of the arthroplasty and the ACDF groups are demonstrated in Table 2. The most frequently injured level was C5/6 (47.6 %) in the arthroplasty group, and C4/5 (40 %) in the ACDF group, respectively.

**Table 2:** Level distributions

Level	No. of level
Arthroplasty (Total level = 21)	
C3/4	5 (23.8 %)
C4/5	4 (19.0 %)
C5/6	10 (47.6 %)
C6/7	2 (9.5 %)
ACDF (Total level = 25)	
C3/4	4 (16.0 %)
C4/5	10 (40.0 %)
C5/6	8 (32.0 %)
C6/7	3 (12.0 %)

### Cervical arthroplasty versus ACDF

Clinical and radiographic outcomes were compared between the two groups (Table 3). The mean operation time between the two groups had no significant difference (214.3 ± 56.8 versus 200.6 ± 58.1 min, *p* = 0.52). The mean estimated blood loss (EBL) also had no significant difference (94.1 ± 102.0 vs. 128.1 ± 125.3 ml, *p* = 0.56) between the two groups. The post-operative range of motion (ROM) at the index level was significantly different (6.2 ± 5.0 vs.0.5 ± 0.4°, *p* = 0.002). The arthroplasty successfully preserved mobility at the indexed level of the cervical spine, whereas ACDF achieved arthrodesis.

**Table 3:** Comparison of the outcome measurements

	Arthroplasty	ACDF	*p* value
	(*n* = 15)	(*n* = 15)	
Operation time (min)	214.3 ± 56.8	200.6 ± 58.1	0.52
Estimated blood loss (ml)	94.1 ± 102.0	128.1 ± 125.3	0.56
Post op neck pain VAS	1.7 ± 2.0	1.0 ± 1.4	0.38
Post op arm pain VAS	1.5 ± 2.1	1.1 ± 1.3	0.63
Post op ROM	6.2 ± 5.0	0.5 ± 0.4	0.002*
Rate of HO	40 % (n = 6)	-	-

*ROM:* range of motion, *HO*: heterotopic ossification

*p < 0.05, statistically significant

**Fig. 1:**
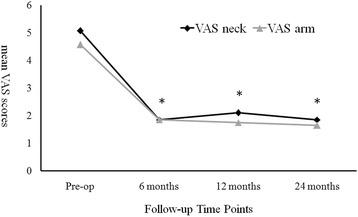
Comparison of mean neck and arm VAS scores in the arthroplasty group (*n* = 15). Significant improvement after surgery was noted for both neck and arm pain at each follow-up time point (i.e. post-operative 6, 12 and 24 months). *Asterisk*, *p*-value < 0.05 compared to pre-operative scores

**Fig. 2:**
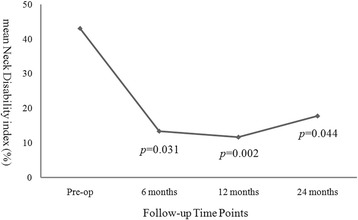
Comparison of mean NDI scores in the arthroplasty group (*n* = 15). Significant improvement after surgery was noted at each follow-up time point (i.e. post-operative 6, 12 and 24 months)

**Fig. 3:**
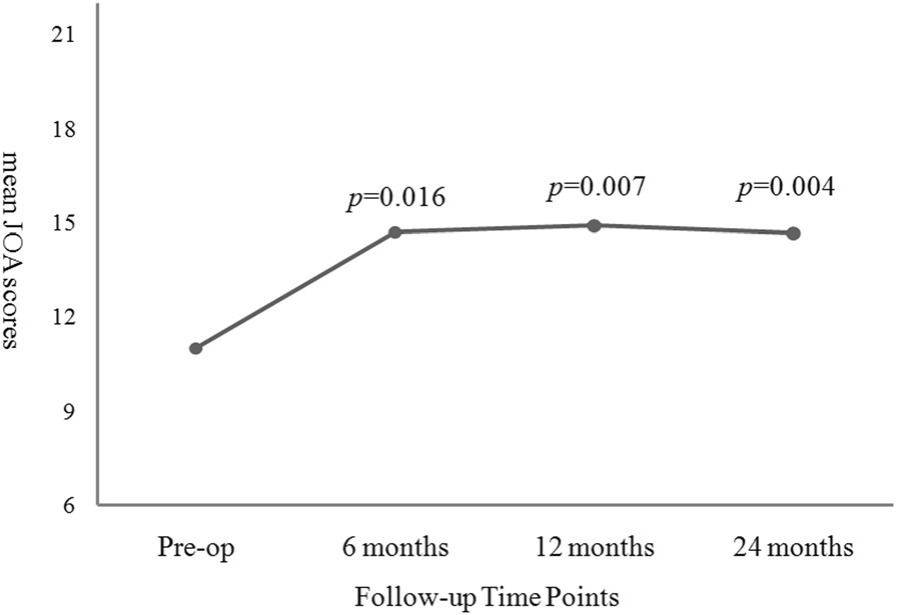
Comparison of mean JOA scores in the arthroplasty group (*n* = 15). Significant improvement after surgery was noted at each follow-up time point (i.e. post-operative 6, 12 and 24 months)

**Fig. 4:**
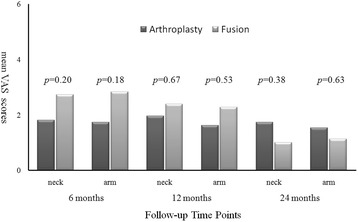
Comparison of mean neck and arm VAS scores between arthroplasty (*n* = 15) and fusion (*n* = 15) groups. No significant difference was noted between the two groups at each follow-up time point (i.e. post-operative 6, 12 and 24 months)

**Fig. 5:**
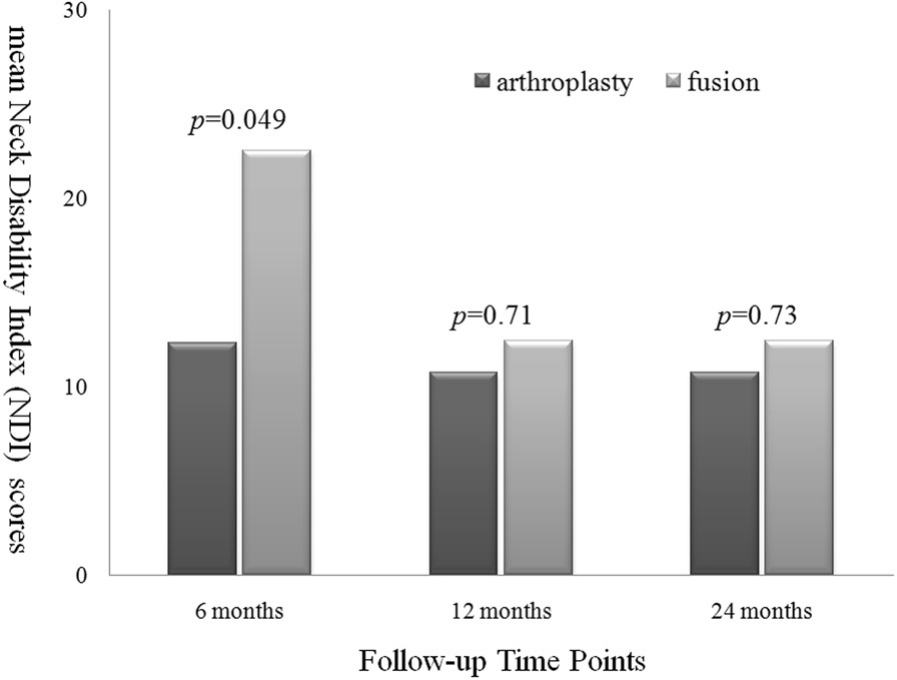
Comparison of mean NDI scores between arthroplasty (*n* = 15) and fusion (*n* = 15) groups. The mean NDI score in the arthroplasty group was better than in the fusion group at post-operative 6 months (*p* = 0.049). No significant difference was noted at other time points (i.e. post-operative 12 and 24 months)

**Fig. 6:**
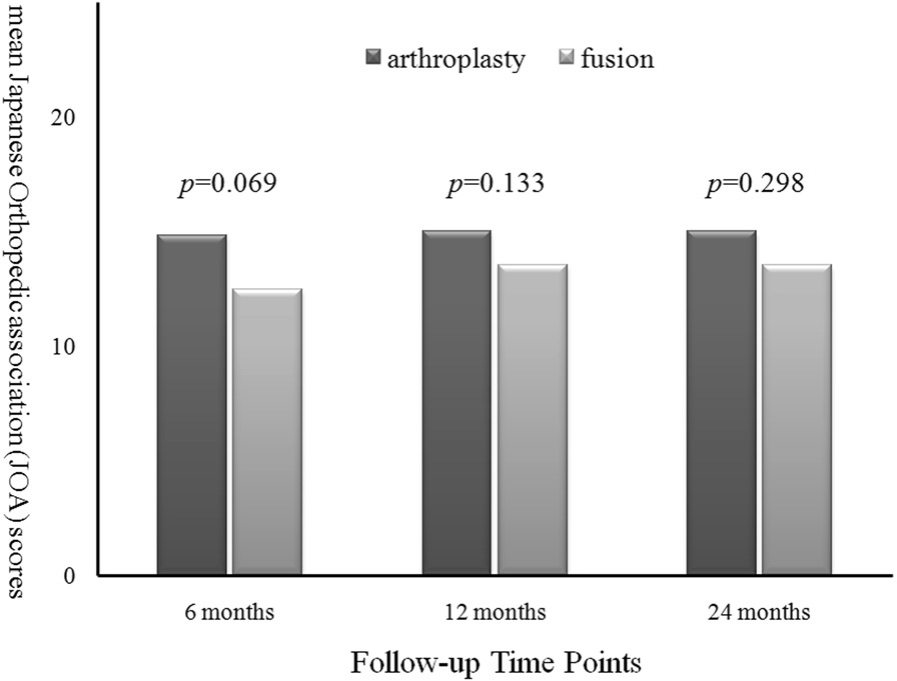
Comparison of mean JOA scores between arthroplasty (*n* = 15) and fusion (*n* = 15) groups. No significant difference was noted between the two groups at each follow-up time point (i.e. post-operative 6, 12 and 24 months)

In the arthroplasty group every parameter of the clinical outcomes, including VAS neck, VAS arm, NDI, and JOA scores, demonstrated significant improvement after the operation when compared to pre-operation (Figs. 1, 2 and 3). The improvement in these clinical outcomes were similar between the arthroplasty group and the ACDF group at 6-, 12- and 24-months post-operation (Figs. 4, 5 and 6), except that the arthroplasty group had significantly better NDI scores than the ACDF group at 6 months post-operation (*p* = 0.049) (Fig. 5).

In the present series of patients there was no secondary surgery (i.e. re-operation, revision, removal of implant, or conversion of arthroplasty to fusion), no surgical complications (i.e. spinal cord injury, permanent dysphagia or hoarseness, wound infection, or post-operative hematoma). In addition, there was no adjacent segment disease (ASD) that required surgery to date.

## Discussion

The FDA trials of cervical arthroplasty enrolled patients with 1- and 2-level cervical disc disease but did not specifically look into the disc herniation caused by minor cervical spinal trauma [1–6, 8, 9]. Since ACDF has been widely accepted as the gold standard surgical approach following neck trauma, those symptomatic patients who require anterior cervical discectomy could definitely be treated by ACDF. However, in selected patients—for example, those young patients who have no spinal cord injury, fracture or instability—cervical arthroplasty may be a viable alternative [23–25]. The present study analyzed 30 patients with traumatic cervical disc herniation who were admitted via the emergency department due to neck injury and who underwent anterior cervical discectomy subsequently. Among these 30 patients, the authors conducted a head-to-head comparison (age- and sex-matched) between cervical arthroplasty and ACDF. Significant improvement in clinical outcomes, including VAS neck, VAS arm, JOA and NDI scores, were demonstrated post-operatively for both the arthroplasty and the ACDF groups. Furthermore, there were little differences in clinical outcomes between the two groups. The demographic characteristics, operation time, and co-morbidities were similar between the two groups. Therefore, the results of this series demonstrate that cervical arthroplasty is a viable option in carefully selected patients with minor cervical trauma. Younger patients who had no spinal cord injury, no bony fracture, and no ligamentous injury causing instability are likely good candidates for cervical arthroplasty.

Cervical spine trauma can range from minor sprain to catastrophic spinal cord injuries [26]. It is widely accepted that ACDF or anterior cervical corpectomy and fusion (ACCF) is the gold standard management procedure for trauma-related C spine injury including HIVD, fracture, locked facet, or dislocation, etc [26–29]. In the instance of some disastrous cases, combined anterior and posterior decompression with instrumentation is imperative for 360° circumferential fixation [30]. The recent literature regarding non-catastrophic traumatic cervical disc herniation without fracture, dislocation or spinal instability were mostly about athletes [26, 28, 29, 31]. The post-operative clinical outcomes were good with ACDF despite some late morbidities of fusion that were reported. Maroon et al. reported two out of five (40 %) patients developed adjacent segment degeneration (ASD) [28]. They also concluded that ASD was an inherent risk after ACDF in a series of 15 traumatic patients [26]. Although there is still inadequate evidence to support the reduction of ASD by cervical arthroplasty, it has been adopted worldwide for more than a decade with excellent results for one- and two-level DDD and spondylosis [3, 5, 6, 9] The best candidates for cervical arthroplasty are young patients with medical refractory radiculopathy caused by soft disc herniation who require anterior cervical discectomy, because cervical arthroplasty relieves neurological symptoms while preserving mobility, and might potentially decrease ASD [24, 25, 32, 33] It is therefore reasonable to infer that patients with mild cervical spinal trauma causing disc herniation (i.e. patients who had no bony fracture or instability but soft disc herniation) can be managed by cervical arthroplasty. However, it must be emphasized that patients with any more severe spinal trauma, for example, severe myelopathy (i.e. Nurick scale 4 or 5) or spinal cord injury worse than ASIA-D, kyphotic deformity, facet incompetence, inadequate integrity of the posterior element, or obvious instability, are definitely contraindicated for arthroplasty.

**Fig. 7:**
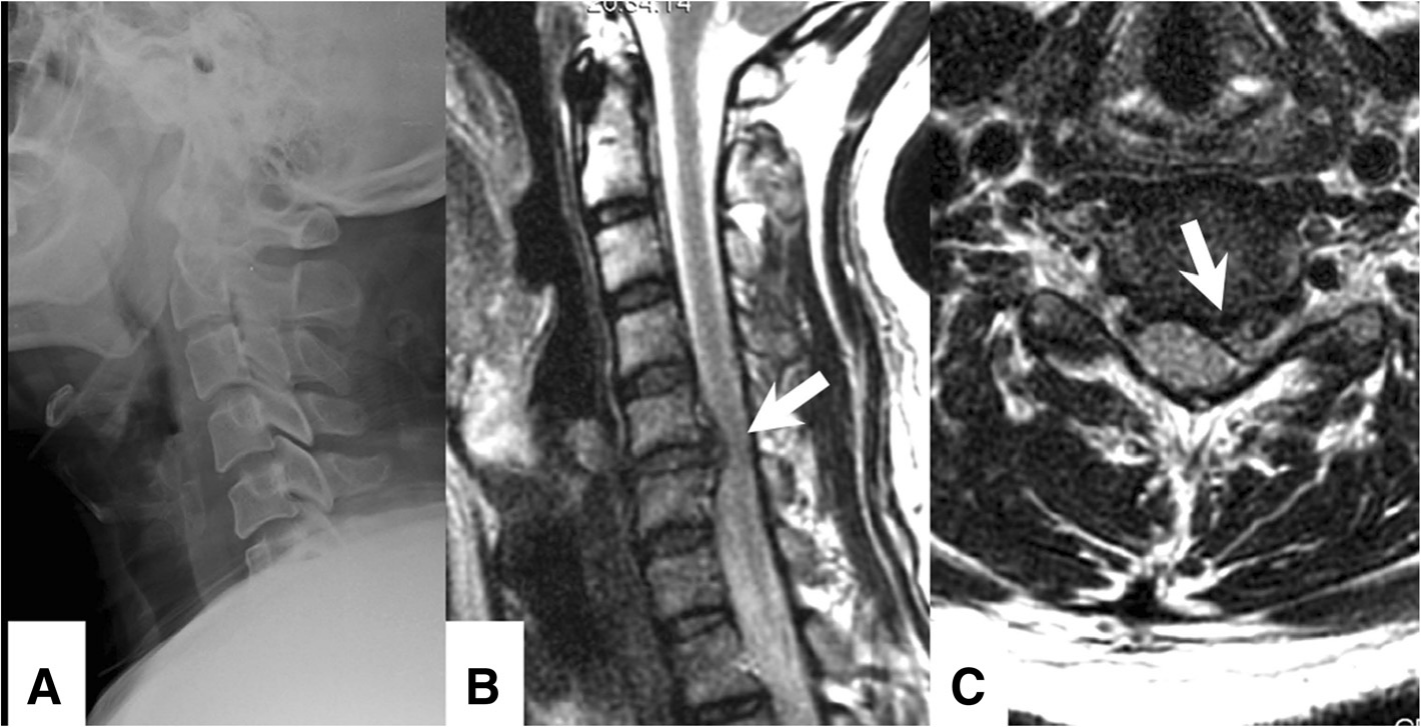
Illustration case of traumatic cervical disc herniation. Pre-operative lateral radiographs (**a**) and MRI T2-weighted image (**b**: sagittal view, and **c**: axial view) of a 46-year-old male with traumatic cervical disc herniation who underwent Prestige LP arthroplasty at C5-6. A trauma-related ruptured disc at left C5-6 level was seen (**b** and **c**, *white arrow*)

**Fig. 8:**
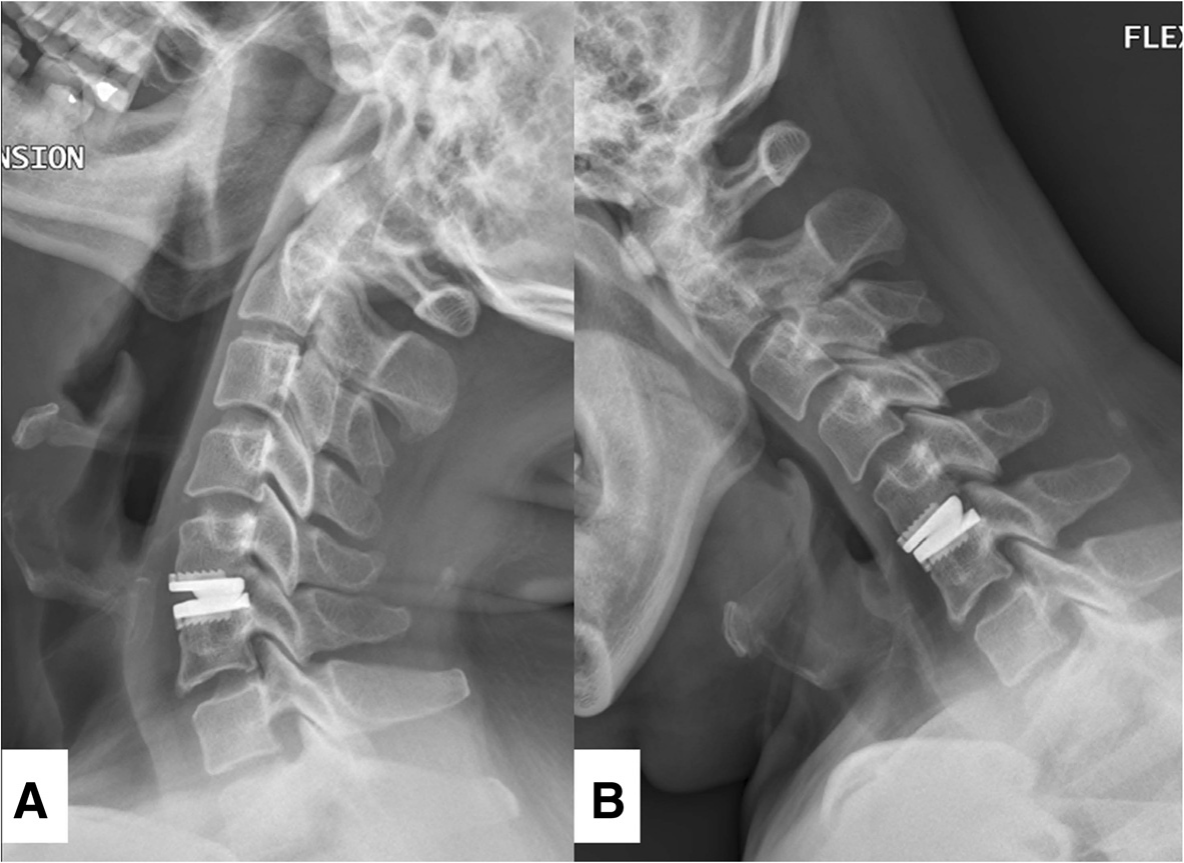
Post-operative dynamic lateral films of the patient mentioned in Fig. 7. The X-ray image demonstrated that the treated levels remained mobile at 24-months follow-up (**a**: extension, and **b**: flexion)

Postoperative stability is a challenge of cervical arthroplasty for trauma. Obviously, patients with fracture or dislocation should not be considered as candidates of arthroplasty. The relative contraindications of cervical arthroplasty also include incompetent posterior elements and ligamentous injury. In the current series, cervical arthroplasty successfully preserved mobility at the indexed level, whereas instrumented ACDF successfully achieved arthrodesis. On dynamic radiographs, the post-operative ROM was individually compared to that of pre-operation at each index level in this study. In the arthroplasty group, the post-operative ROM was preserved when compared to that of pre-operation (6.2 ± 5.0 vs. 5.3 ± 1.6°) (Figs. 7 and 8). This preservation of mobility at each index level of cervical arthroplasty was compatible to the measurement in those FDA trials (approximately 7°) [8]. On the other hand, the intended arthrodesis was achieved in the ACDF group with the application of plate and screw fixation (mean post-operative ROM: 0.5 ± 0.4° versus pre-operation at 7.4 ± 3.6°). The concern for immediate stability after the operation might be an issue initially in the arthroplasty group. However, the patients who received arthroplasty had similar VAS neck pain scores to those who received ACDF at 6-, 12 and 24-months post-operation. Patients in both groups had significant improvement in neck pain. There were no implant failures, migrations, or dislodgements in the current series. Therefore, this result can indirectly attenuate doubts of stability in traumatic disc herniation, under the premises that there was little ligamentous injury.

There were limitations to this study. First, there was inherent selection bias due to the fact that this was a retrospective, non-randomized study. The choices between arthroplasty and ACDF were based on both the surgeons’ and the patients’ preferences. Although both options were equally presented and explained to the patient and family upon consultation at the emergency department, it was possible that the choice could be deviated by economic issues or subjective interpretation. Second, the diagnosis of ligamentous injury for exclusion was not always easy. Patients would have been excluded from the current series if there was evidence of disc rupture and destruction of the anterior longitudinal ligament on the pre-operative magnetic resonance images. Greater than 3.5 mm translation or 20° angular motion found on the flexion-extension lateral radiographs would also have been excluded. However, it was possible that patients with strong neck muscles or moderate spondylosis could limit the detection of laxity or segmental instability after injury. Therefore, the study used the most stringent criteria to include only those trauma cases with minor neck injury. Third, the relatively small sample size could limit the power of this study. Due to the narrow inclusion criteria and at least two years of follow-up, there were not so many cases of minor cervical injury. Nevertheless, the authors compared these specific trauma cases of arthroplasty to the gold standard surgical treatment of ACDF. Moreover, the age- and sex-matched comparison could further reduce the heterogeneity of this cohort.

In summary, this was the first study on the application of cervical arthroplasty in trauma patients who developed disc herniation but little bone and nerve injury. The study could shed light on the indication of cervical arthroplasty for trauma. Future studies are definitely required to corroborate the results and push the envelope of the technology of cervical arthroplasty.

## Conclusion

For selected patients (i.e. no spinal cord injury, no fracture, and no instability) with traumatic cervical disc herniation, cervical arthroplasty yields similar improvement in clinical outcomes to ACDF and preserves segmental mobility.

## Abbreviations

ACDF: Anterior cervical discectomy and fusion
DDD: Degenerative disc disease
FDA-IDE: Food and Drug Administration Investigational Device Exemption
ASIA: American Spinal Cord Injury Association
OPLL: Ossification of posterior longitudinal ligament
VAS: Visual analogue scale
NDI: Neck disability index
JOA: Japanese Orthopedic Association
ROM: Range of motion
ASD: Adjacent segment disease
ACCF: Anterior cervical corpectomy and fusion
HIVD: Herniated intervertebral disc
